# 3D-Printed Biocompatible Frames for Electrospun Nanofiber Membranes: An Enabling Biofabrication Technology for Three-Dimensional Tissue Models and Engineered Cell Culture Platforms

**DOI:** 10.3390/mi16080887

**Published:** 2025-07-30

**Authors:** Adam J. Jones, Lauren A. Carothers, Finley Paez, Yanhao Dong, Ronald A. Zeszut, Russell Kirk Pirlo

**Affiliations:** 1 Chemical and Materials Engineering Department, School of Engineering, University of Dayton, Dayton, OH 45469, USA; ajjones@wakehealth.edu (A.J.J.);; 2 School of Pharmacy, Faculty of Medicine and Health, University of Sydney, Sydney, NSW 2006, Australia; 3 Nonstructural Materials Division, University of Dayton Research Institute, Dayton, OH 45459, USA; ronald.zeszut@udri.udayton.edu; 4 Department of Radiology and Bioengineering, Uniformed Services University of the Health Sciences, Bethesda, MD 20814, USA

**Keywords:** 3D printing, biofabrication, UV-curing resin, microfabrication, tissue engineering scaffold, electrospinning, chitosan, nanofiber membrane, biocompatibility, cell culture, 3D tissue model, barrier model, scaffold handling

## Abstract

Electrospun nanofiber membranes (ESNFMs) are exceptional biomaterials for tissue engineering, closely mimicking the native extracellular matrix. However, their inherent fragility poses significant handling, processing, and integration challenges, limiting their widespread application in advanced 3D tissue models and biofabricated devices. This study introduces a novel and on-mat framing technique utilizing extrusion-based printing of a UV-curable biocompatible resin (Biotough D90 MF) to create rigid, integrated support structures directly on chitosan–polyethylene oxide (PEO) ESNFMs. We demonstrate fabrication of these circular frames via precise 3D printing and a simpler manual stamping method, achieving robust mechanical stabilization that enables routine laboratory manipulation without membrane damage. The resulting framed ESNFMs maintain structural integrity during subsequent processing and exhibit excellent biocompatibility in standardized extract assays (116.5 ± 12.2% normalized cellular response with optimized processing) and acceptable performance in direct contact evaluations (up to 78.2 ± 32.4% viability in the optimal configuration). Temporal assessment revealed characteristic cellular adaptation dynamics on nanofiber substrates, emphasizing the importance of extended evaluation periods for accurate biocompatibility determination of three-dimensional scaffolds. This innovative biofabrication approach overcomes critical limitations of previous handling methods, transforming delicate ESNFMs into robust, easy-to-use components for reliable integration into complex cell culture applications, barrier tissue models, and engineered systems.

## 1. Introduction

Electrospun nanofiber membranes (ESNFMs) have gained significant attention in tissue engineering applications for their ability to mimic natural cellular environments through fibrous architectures that closely parallel the native extracellular matrix (ECM). Their unique morphological characteristics, including extensive specific surface area and controllable pore structures, facilitate essential cellular processes such as attachment, growth, migration, and phenotypic development, establishing them as effective materials for constructing complex three-dimensional tissue models [1]. The adaptability of ESNFMs across diverse polymer systems and their capacity for bioactive compound integration positions them as versatile tools for applications spanning therapeutic delivery systems, regenerative scaffolds [2], and microengineered organ-on-chip (OOC) platforms [3].

These membrane systems demonstrate particular value in developing bilayer barrier tissue constructs [4], where their enhanced permeability characteristics compared to conventional synthetic membranes facilitate improved intercellular interactions and signaling pathways [5]. This property proves especially critical in blood–brain barrier modeling systems that incorporate astrocytes and endothelial cells, where research indicates that spatial proximity between cell populations significantly enhances model fidelity [6]. Previous investigations from our laboratory have demonstrated the benefits of implementing ESNFM substrates in transwell-based blood–brain barrier platforms [7].

The field is currently experiencing a paradigm shift away from conventional static culture systems toward fluid, dynamic, microengineered OOC platforms that incorporate continuous perfusion technologies [8]. While many contemporary microfluidic systems, including commercially available vacuum-mediated strain-inducing platforms [9] developed by Emulate, have achieved significant success, membrane substrate optimization has received limited attention. Despite this gap, research teams across multiple disciplines continue to harness ESNFM technologies to enhance cellular crosstalk in barrier models representing intestinal [10], dermal [11], periodontal [12], and pulmonary [13] tissue interfaces.

Despite these compelling advantages, the widespread adoption of ESNFMs in complex 3D tissue models and engineered bio-devices is significantly hampered by a critical, well-documented limitation: their physical fragility, especially concerning natural polymers [2]. The delicate and friable nature of ESNF biomaterials, especially when dry, poses considerable challenges in harvesting (from the collector), handling, processing, characterization, and device integration. Unsupported ESNFMs are prone to tearing, rolling, folding, and floating, which complicates their use in cell culture and can compromise experimental reproducibility [14]. This difficulty represents a significant bottleneck, limiting the translation of ESNFMs’ vast potential into routine laboratory practice and standardized research tools. To address these challenges, various methodologies have been developed to improve the manipulation and utilization of ESNF mats. These techniques include tape-based methods [15], coating with conductive polymer composites [16], welding [7], gluing, and various clamping strategies [17,18]. Notably, clamping approaches have utilized both bespoke devices and commercially available solutions, such as the CellCrown^TM^ inserts from Scaffdex (Tampere, Finland), which have been employed since 2010 [19]. However, even with these advancements, the scalability of handling techniques remains prevalent. In addition, the scalability of many current handling techniques is often hampered by a reliance on manual operations, which demand significant skill and care [20], consequently introducing variability and limiting overall efficiency and yield.

Beyond basic handling challenges, the integration of ESNFMs into complex engineered systems often requires post-processing steps such as crosslinking, functionalization, and surface metallization for electrical conductivity or sensing applications [21]. These processes further stress the delicate membrane structure, emphasizing the critical need for robust support systems that can withstand various fabrication protocols while maintaining both structural and biological integrity.

Preliminary attempts within our laboratory to support ESNF membranes using conventional fused filament fabrication (FFF) revealed significant technical challenges. Extrusion-based printing of thermoplastic frames using polylactic acid (PLA) and cyclic olefin copolymer (COC) filaments directly onto ESNFMs demonstrated material-dependent success rates. While this approach worked adequately for gelatin ESNFMs, it did not work on ESNFMs incorporating polyethylene oxide (PEO), where poor interfacial adhesion with the thermoplastics led to consistent delamination and failure to achieve mechanical integration.

**Figure 1:**
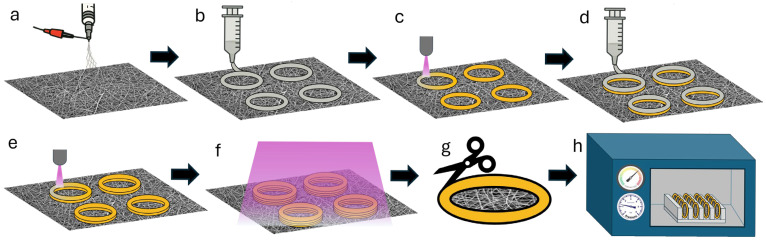
Sequential workflow for electrospun nanofiber membrane (ESNFM) framing fabrication. (**a**) Electrospinning of nanofibers onto non-stick aluminum foil collector substrate; (**b**) following transfer to printer build plate, initial layer of UV-curable resin rings is extruded via syringe-based deposition; (**c**) UV pen system traces and photocures the deposited resin rings; (**d**) subsequent resin layer is printed using identical extrusion parameters; (**e**) UV pen performs secondary tracing and curing cycle to complete layer-by-layer fabrication; (**f**) completed 4-layer ring structures undergo comprehensive post-print UV-curing protocol; (**g**) excess ESNFM material is precision-trimmed from cured, framed samples; (**h**) trimmed ring assemblies are subjected to one of two cleaning and extended curing protocols for biocompatibility enhancement.

To address these pervasive challenges, we introduce a novel on-mat framing technique inspired by UV-assisted Direct Ink Writing (UV-DIW) principles (Figure 1), adapted for laboratories with extrusion-based systems, but without specialized immediate curing equipment. Direct Ink Writing, as an extrusion-based additive manufacturing technique, fabricates three-dimensional structures through the precise deposition of viscoelastic inks from a nozzle [22,23]. While conventional DIW requires materials with specific rheological properties, particularly shear-thinning behavior that enables flow through the nozzle followed by immediate shape retention upon deposition [23,24], many high-performance photocurable resins possess low initial viscosities that preclude self-supporting behavior [25]. Optimally, UV-assisted DIW employs in situ ultraviolet irradiation during the extrusion process, facilitating immediate photopolymerization that prevents resin spreading and enables precise feature definition.

This manuscript details the development, characterization, and validation of this enabling biofabrication method. We demonstrate successful fabrication of biocompatible circular frames using Biotough D90 MF on chitosan–polyethylene oxide (PEO) ESNFMs, show improved handling characteristics and structural integrity of framed ESNFMs, validate their ability to withstand post-processing steps, and, crucially, establish biocompatibility through comprehensive extract and direct contact assays. The objective of this work is to present a practical and effective solution that significantly enhances the handling, processing, and application of ESNFMs, thereby facilitating their broader use in advanced cell culture, 3D tissue model development, and integration into engineered biological systems.

## 2. Materials and Methods

### 2.1. Materials

Ethanol (200 proof, ACS/USP grade) was obtained from Decon Labs, Inc. (King of Prussia, PA, USA). Phosphate-buffered saline (PBS, pH 7.4), dopamine hydrochloride, penicillin–streptomycin, GlutaMAX supplement, Blasticidin S HCl, G418 sulfate (Geneticin), and isopropyl alcohol were purchased from Thermo Fisher Scientific (Waltham, MA, USA). Chitosan, CS (low molecular weight, Sigma-Aldrich, Saint Louisan, MO, USA, 448869-50G), acetic acid (glacial, ACS reagent, ≥99.7%, Product No. 320099), Poly (ethylene oxide), PEO (average MW~2,000,000, powder), and Tris buffer were obtained from Sigma-Aldrich (St. Louis, MO, USA). Gold sputter coating targets (99.99% purity) were used for SEM sample preparation. Polydopamine (PDA) and Tris buffer were obtained from Sigma-Aldrich. Telomerase-Immortalized Human Microvascular Endothelial (TIME-GFP) cells, expressing green fluorescent protein (GFP), and recommended cell culture media components, including ATCC-formulated Vascular Cell Basal Medium and Endothelial Cell Growth Kit-VEGF components, were obtained from ATCC (Manassas, VA, USA). Penicillin–streptomycin, GlutaMAX supplement, Blasticidin S HCl, and G418 sulfate (Geneticin) were purchased from Thermo Fisher Scientific (Waltham, MA, USA) or ATCC. Laboratory-grade PVC tubing (3/8″ ID, 1/2″ OD; ZUSA-HT-1784, USASEALING) was used for manual stamping tools.

The selection of an appropriate UV-curable resin was critical to the success of the extrude-and-cure printing approach. Initial attempts utilized a quick-set epoxy resin (JB Weld 5 min epoxy, JB Tools, Livonia, MI USA); however, this material presented significant limitations. The progressive curing characteristics severely restricted print time and compromised printability, while subsequent biocompatibility testing revealed cytotoxic properties that precluded biomedical applications. Additionally, the epoxy’s viscosity profile and curing kinetics proved incompatible with the precise control required for UV-DIW processing.

To address these limitations, Biotough D90 MF (Monomer Free) was selected based on its exceptional mechanical and biocompatibility specifications [26] that directly address the requirements for ESNFM stabilization applications. The resin is specifically engineered for Class I, II, and III biomedical devices and formulated to meet stringent regulatory standards, including FDA, ISO 20795 [27], ISO 13485 [28], and ISO 10993 [29,30] compliance requirements. The monomer-free formulation reduces potential leachable toxicity concerns, directly supporting biocompatibility requirements for tissue engineering applications. Additionally, the manufacturer’s specified properties for the resin include tensile strength >50 MPa and ultra-high flexural strength of 110–130 MPa, providing the structural integrity necessary for robust frame fabrication [31]. The high rigidity (Young’s modulus 2700–3300 MPa) and Shore hardness rating of D87-90 indicate excellent dimensional stability and surface durability, respectively, suggesting suitability for inclusion in clamped bioreactor designs [32,33].

Telomerase-Immortalized Human Microvascular Endothelial cells expressing green fluorescent protein (TIME-GFP) were selected for this study based on their successful use in our previous barrier models and their established role as a sensitive indicator for biocompatibility. The GFP allowed us to observe cell morphology and proliferation of cells despite the obscuring effects caused by the ESNFMs under brightfield microscopy. The expression of GFP also permitted observation of samples throughout the 7-day culture period using fluorescent microscopy. Furthermore, as the cells lining the vasculature, endothelial cells act as critical “sentinels” that are the first to encounter and respond to any leachable substance that enters the systemic circulation, making them a highly sensitive and relevant model for foundational biocompatibility assessment [34].

The selection of chitosan–polyethylene oxide (CS:PEO) as the model electrospun system for this work was guided by multiple considerations critical for biomedical applications. Chitosan provides inherent biocompatibility, antimicrobial properties, and established efficacy in wound healing applications, while demonstrating structural similarity to glycosaminoglycans found in the native ECM. The incorporation of PEO serves exclusively as a processing aid to achieve stable electrospinning through viscosity modification and chain entanglement optimization. In our preliminary work, we developed a working solution that minimized PEO content to the lowest concentration (6% *w*/*w*), which supported consistent and uniform fiber formation. We also observed (as have others [35]) that PEO dissolution in aqueous cell culture media induces membrane swelling and “billowing” behavior. This dimensional instability, caused by PEO’s hydrophilic dissolution, creates stress concentrations that render ESNFMs susceptible to tearing during routine culture manipulations. The optimized 94:6 CS:PEO ratio represents the minimum PEO content required for consistent fiber formation while maintaining dimensional stability in aqueous environments without further crosslinking. This formulation preserves chitosan’s biological functionality and structural integrity throughout the culture period, making it an ideal candidate system for validating the framing technology across diverse tissue engineering applications, including barrier models and wound healing scaffolds.

### 2.2. Electrospinning of Chitosan–PEO Nanofiber Membranes (ESNFMs)

#### 2.2.1. Preparation of Electrospinning Solution

A chitosan–PEO 94:6 *w*/*w* polymer blend was dissolved in 4:1 acetic acid–water at a total polymer concentration of 1.7% *wt*/*v*. First, 640 mg of CS and 32 mL of acetic acid were added to a 60 g-capacity Flacktek cup and shaken for 10 s, after which 8 mL of DI water was added and the entire solution was shaken again before magnetically stirring for 4 days at 60 °C and 100 rpm. On the 4th day, 32 mg PEO was added, and the solution was shaken by hand for 10 s and allowed to sit at 60 °C overnight. On the (fifth) day of use, the solution was mixed at 1000 rpm for 1 min in a centrifugal mixer (Flakteck), magnetically stirred at 60 °C and 100 rpm for 1 h, and then mixed again for 1 min at 1000 rpm in the centrifugal mixer.

#### 2.2.2. Electrospinning Process

Electrospinning was performed in open air, with a room temperature between 24 and 26 °C, and relative humidity between 20% and 40%. The ES working solution was loaded into a 5 mL plastic BD syringe connected to 1.5″ blunt 27 Gauge needle (ID 0.08″, OD 0.016″). The flow rate of the solution was 0.4 mL/h, at a fixed tip-collector distance of 25 cm. An alternating potential of ±15 kV was applied with a polarity switching frequency of 60 s^−1^ over a period of 4 h by a high-voltage power supply. The power supply (K12-30R, Matsusada Precision Inc., Shiga, Japan) was controlled with a computer running a custom VBA program through an optically isolated interface (CO-HV, USB-OPT, Matsusada Precision Inc.). The nanofibers were collected on grounded non-stick aluminum foil (Reynolds Wrap, Reynolds Consumer Products, Lake Forest, IL, USA), and dried for at least 12 h in a desiccator box (<10% humidity).

#### 2.2.3. Transfer of Electrospun Mat to Build Plate

After collection on non-stick aluminum foil, the mat and foil were transferred to a 6 × 6 × 1/4″ glass plate. The plate was sprayed with Aqua Net hairspray (Lornamead Inc., Tonawanda, NY, USA), and the mat was placed foil-side down onto the glass plate and carefully laid out to avoid wrinkles. A second non-stick foil sheet was applied on top of the nanofiber mat, with the non-stick side facing the mat, and the mat was adhered by smoothing from the center outwards toward the edges with a cardboard edge. The smoothing foil was then removed, resulting in a flat surface for printing.

### 2.3. Design and Biofabrication of Support Frames

Support frames were designed as circular structures with a target outer diameter of 13 mm, a frame width of 1.6 mm, and a height of 0.8 mm. Additional solid nanofiber/resin composite disks with a target diameter of 13 mm and 0.8 mm height were also fabricated for biocompatibility studies. These geometries were chosen to be compatible with standard 24-well cell culture plates, allowing the framed ESNFM to function as a suspended scaffold. Frame designs were created using Inventor (Autodesk, San Rafael, CA, USA) and exported as STL files.

#### 2.3.1. Three-Dimensional Printing Method

Three-dimensional printing of circular frames directly onto ESNFMs (supported on their glass plate substrate) was performed using a Hyrel 3D Engine HR printer equipped with an SDS5 syringe extruder fitted with a 17-gauge ½″ blunt needle and a UVP-365 UV Light Pen head. The printer was loaded with Biotough D90 MF resin in the syringe extruder. Prusa slicer was used to generate the G-code with the parameters listed in Table 1. Control of the Hyrel tools required custom G-code for the filament/extruder, which is included in the Supplementary Materials.

#### 2.3.2. Manual Stamping Method

**Figure 2:**
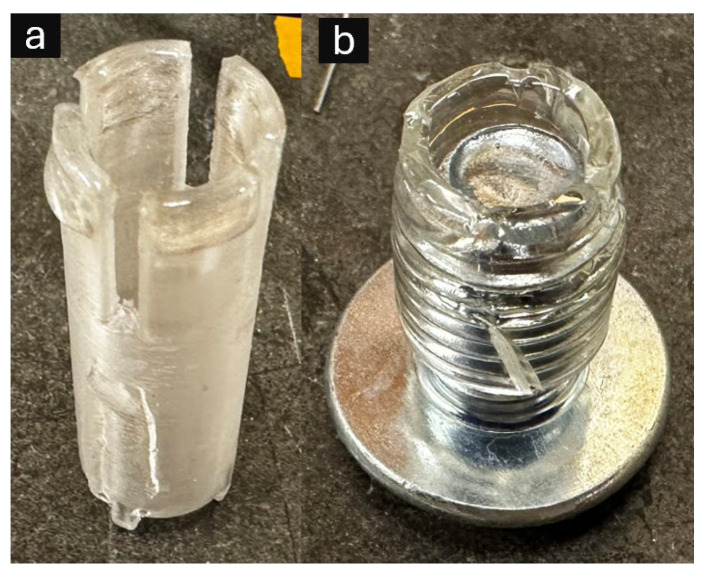
Alternative manual stamping devices for resin frame fabrication: (**a**) 3D printed cyclic olefin co-polymer (COC) stamping tool featuring precisely engineered circular geometry with integrated air release channels to prevent bubble formation during resin transfer; (**b**) laboratory-grade polyvinyl chloride (PVC) tubing (1/2″ outer diameter, 3/8″ inner diameter) with inserted bolt to maintain consistent circular configuration and triangular cutouts for air displacement control.

As a simpler alternative to 3D printing, circular frames were fabricated using a manual contact stamping method. Two types of stamping tools (Figure 2) with target dimensions of 13 mm outer diameter and 0.8 mm frame width were developed: (1) 3D printed using COC filament and (2) fabricated from 1/2″ outer diameter laboratory-grade PVC tubing with slots cut into the stamping face to prevent air bubble formation during lifting.

Stamping process:

- A well of a 24-well plate was filled with Biotough D90 MF resin.
- The stamping tool was brought into contact with the resin surface to load its stamping face.
- The tool was removed and brought into gentle contact with the ESNFM.
- A slight rotation (approximately 90 degrees) was applied to help impart a continuous circular deposit.
- Stamped resin was pre-cured with a 385–395 nm 35 W flashlight (uvBEAST V3). Each ring was exposed for approximately 5 s at a distance of 2 inches.
- The ESNFM with applied resin frame underwent the same post-curing process as the 3D printed samples.

### 2.4. Post-Fabrication Processing and Purification

Following either 3D printing or manual stamping, comprehensive post-processing was performed to ensure complete curing and biocompatibility.

#### 2.4.1. Initial Post-Curing and Frame Separation

Final UV curing was performed with a PortaRay 400R UVA Light curing system (UViTRON International) with a 400 W lamp emitting 365 nm over a 5 × 5″ area. Foil-backed mats with pre-cured rings were exposed three times for 10 s with a 30 s cooling period between exposures. The rings were then removed from the foil by slowly dragging the taught foil backing over the 90° edge of a table/bench. Rings were then trimmed and placed in a slotted tray designed for the purpose of handling framed mats.

#### 2.4.2. Cleaning and Extended Curing Protocols

To remove unreacted resin residues and ensure biocompatibility, framed ESNFMs and composite disks were subjected to one of two processing protocols:

Protocol A (24): Vacuum (11 Kpa) oven baking for 24 h at 60 °C.
Protocol B (48): Vacuum oven baking for 24 h at 60 °C, followed by soaking in isopropyl alcohol for 1 h, brief rinse in fresh isopropyl alcohol for 1 min, and second vacuum oven baking for 24 h at 60 °C.

#### 2.4.3. Surface Treatment and Disinfection

Framed NF mats were neutralized by soaking once overnight in a 1 M aqueous solution of K_2_CO_3_, mixed 30:70 with ethanol [35], and again in fresh solution for 1 h. Samples were then rinsed 3 times for 10 min each in DI water. Neutralized samples were then placed in a 24-well plate. Next, sample surfaces were modified to promote cell adhesion with polydopamine (PDA) [36] by covering with 1 mL of the PDA solution and soaking for 1 h. The PDA solution was freshly prepared before use to reduce particle size and precipitation by dissolving 0.5 mg/mL of dopamine hydrochloride in a Tris-HCl buffer solution (0.01 M, pH 8.5). The PDA solution was aspirated, and the samples were rinsed 3 times with phosphate-buffered saline. In the final preparation step before cell seeding, samples were disinfected by soaking in 70:30 ethanol–water for 30 min and allowed to dry under UV in a biosafety cabinet for 30 min.

### 2.5. Material Characterization

#### Scanning Electron Microscopy (SEM)

The morphology of ESNFMs (fiber diameter and porosity) and the structure of the 3D printed/stamped circular frames, including the ESNFM–frame interface, were examined using SEM(Phenom ProX Desktop PW-100-017, Thermo Fisher Scientific, Eindhoven, The Netherlands). Dry samples were sputter-coated with approximately 5 nm of gold using a Denton Vacuum Desk V Sputter Coater for 60 s at 20 mA. Images were acquired at various magnifications using 15–20 kV accelerating voltage. Fiber diameter was measured from SEM images using the SIMPoly [37] Matlab (2024a) method and ImageJ (1.54p) [38] software, for validation of SIMPoly analysis, counting 20 fibers per membrane.

### 2.6. Cell Culture and Biocompatibility Assessment

#### 2.6.1. Cell Culture Conditions

TIME-GFP cells were cultured in T75 flasks with Vascular Cell Basal Medium (ATCC PCS-100-030) supplemented with Endothelial Cell Growth Kit-VEGF (ATCC PCS-100-041), 1% Penicillin–streptomycin, 1X GlutaMAX supplement, Blasticidin S HCl (12.5 µg/mL), and G418 sulfate (200 µg/mL). Cells were maintained at 37 °C in a humidified 5% CO_2_ incubator and passaged upon reaching 70–80% confluency. On Day 0 of experiments, cells were dissociated with trypsin/EDTA, neutralized with trypsin neutralizing solution, and centrifuged at 200× *g* for 6 min, before resuspension in culture medium.

#### 2.6.2. Biocompatibility Testing Design

Biocompatibility evaluation employed a dual-platform assessment strategy incorporating both extract-based and direct contact methodologies in accordance with ISO 10993-5:2009 standards [30]. Testing compared two UV-curing protocols (24 h vs. 48 h) to establish optimal processing parameters. All biocompatibility experiments were conducted with a minimum of 4 biological replicates for each test condition (e.g., format, orientation, and processing protocol).

Extract Testing Protocol: Resin extracts were prepared using standardized extraction methodology with culture medium (37 °C, 24 h incubation) following ISO 10993-12:2021 guidelines [39]. TIME-GFP cells were seeded at 20,000 cells per well in 24-well plates, representing the optimal linear range for the CCK-8 assay (R^2^ = 0.9973). Cell viability was assessed at Day 1 using the Cell Counting Kit-8 (CCK-8; APExBIO, Houston, TX, USA) according to the manufacturer’s instructions.

Direct Contact Assessment: Surface biocompatibility evaluation implemented optimized seeding density (100,000 cells per well) to ensure adequate analytical sensitivity for detecting material-specific responses on complex nanofiber architectures. The assessment included multiple configurations:

- Circular frames with varied orientations (frame up vs. frame down);
- Composite disks with varied surface exposure (nanofiber side up vs. resin side up);
- Control surfaces (standard tissue culture plastic).

Cell viability was assessed using the CCK-8 assay at Day 7, with morphological assessment via phase-contrast and fluorescence microscopy on Days 1, 3, 5, and 7.

#### 2.6.3. Statistical Analysis and Quality Control Framework

Quantitative biocompatibility data analysis employed a comprehensive statistical framework designed for regulatory compliance and methodological rigor. Cellular responses were normalized to contemporary control viability within each experimental plate using plate-specific normalization protocols to eliminate inter-assay enzymatic variability inherent in CCK-8 analysis. This variability, which can reach 15–20% between plates due to reagent batch differences, enzyme storage conditions, and minor temperature fluctuations during incubation, necessitates rigorous normalization strategies. By expressing the results as the percentage of control within each plate, the methodology provides direct biological interpretation while maintaining statistical independence across experimental batches and eliminating systematic bias from enzymatic activity variations.

Quality Control and Precision Standards: Technical precision was evaluated using coefficient of variation analysis, with experimental units exhibiting CV > 15% between technical replicates excluded from analysis. This stringent criterion, aligned with FDA bioanalytical validation standards, ensured measurement reliability exceeding conventional biocompatibility assessment protocols.

Primary Statistical Analysis: Biocompatibility assessment employed one-way ANOVA with post hoc Tukey HSD multiple comparisons for extract evaluation, and mixed-effects modeling for direct contact assessment accounting for format and orientation dependencies. Plate-specific random effects were incorporated to address the hierarchical experimental structure. Effect sizes were calculated using Cohen’s d with pooled standard deviation methodology, supplemented by meta-analytic synthesis employing inverse-variance weighting to combine effect estimates across assessment platforms.

Regulatory Compliance Validation: Biocompatibility threshold testing implemented one-sample *t*-tests against a 70% viability criterion established by ISO 10993-5 standards, with safety margin quantification through confidence interval assessment. Statistical significance was set at α = 0.05 for all analyses.

### 2.7. Proof-of-Concept: Electroless Gold Coating Process

Note: This proof-of-concept investigation was conducted during initial method development using preliminary JB Weld 5 min epoxy frames, prior to optimization with Biotough D90 MF resin. While the demonstration validates the general concept of subjecting framed ESNFMs to aggressive multi-step chemical processing, specific adhesion characteristics and chemical resistance may differ between epoxy and UV-curable resin systems. The protocol is presented to illustrate the enabling nature of frame support for complex post-processing applications that would be impractical with unsupported membranes.

#### 2.7.1. Copper Deposition Process

ESNFMs were soaked in an aqueous CuSO_4_ solution (approximately 0.5 M, certified ACS, Thermo ScientificChemical, Waltham, MA, USA) at 40 °C for 10 min. This process resulted in a blue coloration on the ESNFMs, indicating that the fibers had absorbed the cupric ion from solution. The absorbed cupric ion was then reduced by immersion of the samples into 3 g/L NaBH_4_ (99%, Acros Organics, Thermo Scientific Chemicals, Waltham, MA, USA) at 40 °C for 5 min [40]. Occasional flipping of samples in the reducing chemistry was required to release gas bubbles which accumulated on the samples due to the generation of hydrogen gas in the redox reaction. This process resulted in a brown coloration of the samples, indicating a reduction in the absorbed copper. Samples were then soaked in DI water for approximately 5 min to remove any additional NaBH_4_.

#### 2.7.2. Synthesis of Gold–Tiopronin Complex

Due to gold’s low solubility in typical aqueous solutions, tiopronin (C_5_H_9_NO_3_S) was used as a complexing agent due to its effectiveness and non-toxicity [41]. This was achieved by first preparing a gold–tiopronin complex. A tetrachloroauric acid solution was prepared by dissolving approximately 1 g of gold shot (99.95%, Thermo Scientific Chemicals, Waltham, MA, USA) in a mixture of 3.75 mL hydrochloric acid (certified ACS, Fisher Chemical) and 1.25 mL nitric acid (certified ACS, Fisher Chemical). Separately, a second solution was prepared containing 2.75 g tiopronin (97%+, Tokyo Chemical Industry (TCI) (America, Portland, OR, USA) and 1.5 g glacial acetic acid (certified ACS, Fisher Chemical) diluted to 100 mL with DI water. The tetracholoauric acid solution was added to this second solution, yielding a crude gold–tiopronin complex, which was isolated by centrifuging. The crude gold–tiopronin complex was purified by dissolving in an aqueous solution containing 0.35 g K_2_CO_3_ (ACS, Thermo Scientific Chemicals) and 0.083 g tiopronin diluted to 100 mL with DI water. The solution was then adjusted to pH 2 using glacial acetic acid before being centrifuged again. The precipitate was then washed in 100 mL DI water before final centrifuging to yield the purified gold–tiopronin complex, which was then dried at 80 °C. Note: All chemical amounts listed here are based on an initial gold mass of 1 g and were scaled proportionally with the actual amount of gold used for each initial tetracholoauric solution.

#### 2.7.3. Gold Deposition Process

The gold electroless plating solution consisted of 1.8 g gold–tiopronin complex, 0.1 g 2-Amino-5-mercapto-1,3,4-thiadiazole (98%+, TCI America), 0.75 g potassium citrate (USP/FCC, Fisher Chemical), 0.75 g potassium phosphate (99%+, Fisher BioReagents, Waltham, MA, USA), 0.25 g potassium triphosphate (94%+, Strem, Newburyport, MA, USA), 0.25 g ethylenediaminetetramethylenephosphonic acid (98%+, TCI America), 5.0 mg polyethylene glycol 1000 (Thermo Scientific Chemicals), and 1.0 g ascorbic acid (certified ACS, Fisher Chemical) in DI water, adjusted to pH 6 using sulfuric acid (certified ACS, Fisher Chemical) and potassium hydroxide (certified ACS, Fisher Chemical), and diluted to 50 mL. The copper-coated ESNFMs were then immersed in the gold plating solution at 70 °C for 10–60 min for the desired thickness. After plating, the ESNFMs were gently rinsed in DI water and left to try at room temperature. Note: Gold coating was demonstrated with preliminary frame materials during method development and was not repeated with the optimized Biotough D90 MF resin system.

## 3. Results

### 3.1. ESNFM Framing

**Figure 3:**
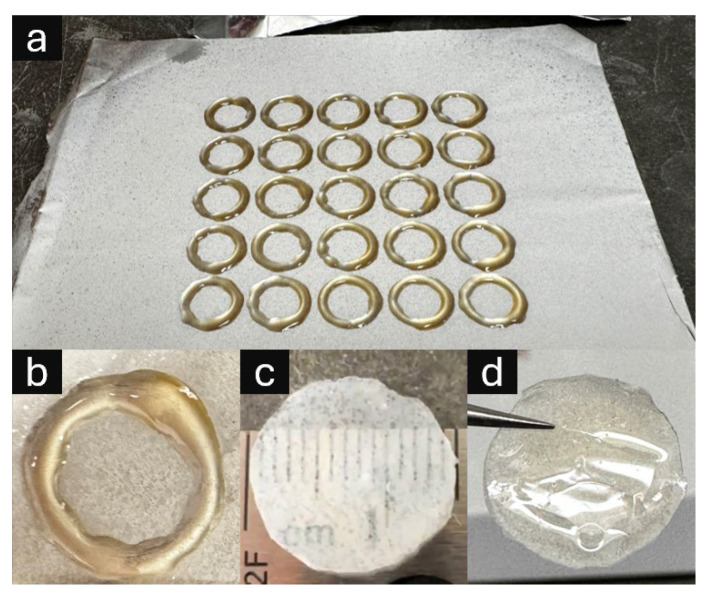
Three-dimensional printed UV-curable resin support structures integrated with electrospun nanofiber membranes (ESNFMs). (**a**) Five-by-five array of circular frames directly printed onto ESNFM substrate, demonstrating scalable fabrication and consistent frame–membrane integration; (**b**) close-up of resin ring on ESNFM; (**c**) nanofiber/resin composite disk oriented with ESNFM surface exposed (nanofiber side up), revealing embedded fibrous texture within resin matrix; (**d**) composite disk oriented with pure resin surface exposed (ESNFM side down), showing smooth polymer interface for comparative biocompatibility assessment.

Despite target models specifying a 13 mm diameter, the fabricated parts measured approximately 15 mm due to resin spreading during the layer-by-layer printing and curing process. The fabrication time for arrays of 25 samples was approximately 5 min, equating to ~12 s per layer per circular frame before entire layer curing. This scalable fabrication approach enabled direct integration of 3D printed support structures with electrospun nanofiber membranes, as demonstrated in Figure 3.

The integrated frame–membrane composites exhibit distinct morphological characteristics depending on orientation. Figure 3 illustrates both the fabrication scalability through five-by-five circular frame arrays and the resulting composite material properties. The resin successfully infiltrates the nanofiber matrix during printing, creating a dual-surface composite with contrasting interfaces: one surface exposing the embedded fibrous texture within the resin matrix, and the opposing surface presenting a smooth polymer interface suitable for comparative biocompatibility assessment.

### 3.2. ESNFM Characterization and Frame Integration

**Figure 4:**
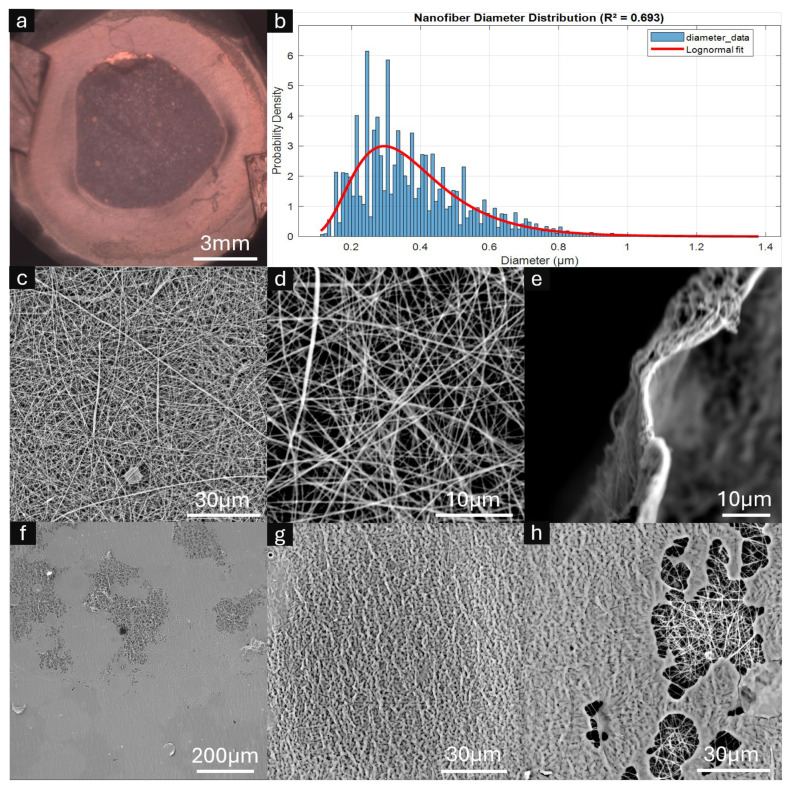
Characterization of framed electrospun nanofiber membranes (ESNFMs). (**a**) Optical microscopy image showing overall frame–membrane integration; (**b**) Nanofiber diameter distribution histogram with fitted lognormal curve (mean = 381 nm, mode = 295 nm, R^2^ = 0.693); (**c**,**d**) high-magnification SEM images revealing characteristic nanofiber mat architecture and porosity; (**e**) cross-sectional view of ESNFM edge demonstrating mat thickness <5 μm; (**f**–**h**) SEM analysis of ESNFM underside at the resin–nanofiber interface. Resin penetration was heterogeneous, with incomplete saturation observed in regions (**f**,**h**), while areas of successful resin integration retained characteristic nanofiber surface texture imparted by the underlying fibrous architecture (**g**).

Comprehensive morphological characterization of the integrated frame–membrane composites was performed using scanning electron microscopy (SEM) and optical imaging techniques, with the results presented in Figure 4. SEM analysis revealed uniform chitosan–PEO fibers without beading or melting artifacts, demonstrating successful electrospinning parameters. Quantitative fiber diameter analysis via SIMPoly yielded amean diameter of 381 nm with a standard deviation of 165 nm and a modal diameter of 295 nm, as illustrated in the diameter distribution histogram (Figure 4b). A lognormal distribution provided an excellent fit to the data (R^2^ = 0.693), which is characteristic of electrospun nanofibers and indicates consistent process control. The right-skewed distribution with median diameter of 350 nm reflects the stochastic nature of the electrospinning processThis automated analysis was validated with 20 manual measurements (297 ± 35 nm) made using the ImageJ line tool [42] confirming the accuracy of the SIMPoly characterization method.

Cross-sectional examination revealed an estimated mat thickness of 1–2 µm, with high-magnification SEM imaging confirming the characteristic nanofiber mat architecture and inherent porosity (Figure 4c,d). The overall frame–membrane integration achieved uniform structural consistency across the composite surface (Figure 4a).

Critical assessment of the resin–nanofiber interface revealed heterogeneous penetration behavior at the ESNFM underside. While some regions exhibited incomplete resin saturation through the mat thickness (Figure 4f,h), successful integration zones retained the characteristic nanofiber surface texture imparted by the underlying fibrous architecture (Figure 4g). This heterogeneous resin penetration pattern reflects the complex interplay between printing parameters, resin viscosity, and nanofiber mat porosity during the layer-by-layer fabrication process.

### 3.3. Biocompatibility Assessment Results

Comprehensive biocompatibility evaluation demonstrated systematic enhancement through processing optimization and format-dependent performance differences (Table 2). Statistical analysis revealed significant processing parameter effects across both assessment methodologies.

#### 3.3.1. Extract Biocompatibility: Processing Parameter Optimization

Extract testing demonstrated robust biocompatibility with systematic enhancement through extended UV-curing protocols (F_2,__21_ = 17.55, *p* < 0.0001). The 48 h versus 24 h comparison yielded a 26.2 percentage point improvement (95% CI: 14.9–37.4 points, *p* < 0.0001), with an exceptional effect size magnitude (Cohen’s d = 2.91). Both processing conditions significantly exceeded biocompatibility thresholds, with extended curing providing a 66% safety margin above regulatory requirements.

#### 3.3.2. Direct Contact Biocompatibility: Format and Configuration Effects

Direct contact assessment revealed processing-dependent biocompatibility enhancement (49.8% to 67.9%, *p* = 0.042, Cohen’s d = 0.82) with significant format-specific differences. Disk configurations provided superior cellular support compared to ring geometries (78.2% vs. 54.3%, *p* = 0.028), indicating geometry-dependent biocompatibility mechanisms. The optimal performance combination (disk format with 48 h processing) achieved Grade 1 biocompatibility classification suitable for tissue engineering applications.

### 3.4. Morphological Assessment

**Figure 5:**
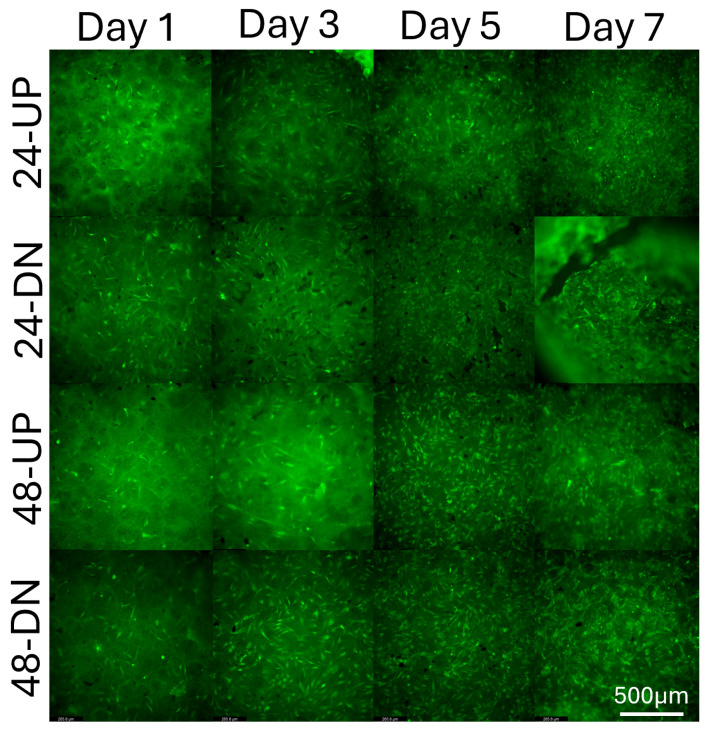
Temporal progression of TIME-GFP endothelial cell growth and integration on framed electrospun nanofiber membranes (ESNFMs). Fluorescence microscopy time-course analysis (Days 1, 3, 5, and 7) demonstrating cellular attachment, proliferation, and spatial distribution on UV-curable resin frames subjected to different processing protocols. Experimental conditions compare 24 h versus 48 h vacuum oven baking treatments, with framed ESNFMs oriented either resin-side-up (UP, exposing nanofiber surface to cells) or resin-side-down (DN, positioning frame below ESNFM). Green fluorescent protein (GFP) expression enables visualization of viable endothelial cells, revealing progressive cellular colonization and network formation across the nanofiber architecture. Notable observations include enhanced cellular density and improved spatial distribution with extended processing protocols (48 h treatment) and superior cell attachment characteristics in the resin-side-up configuration. Imaging parameters: fluorescence microscopy with GFP filter set, consistent exposure conditions maintained across all time points for quantitative comparison.

Comprehensive temporal analysis of cell–substrate interactions was conducted using fluorescence microscopy to evaluate TIME-GFP endothelial cell biocompatibility with framed ESNFM surfaces, as presented in Figure 5. The experimental design incorporated systematic evaluation of processing variables, including vacuum oven baking duration (24 h versus 48 h treatments) and substrate orientation (resin-side-up versus resin-side-down configurations) to optimize cellular attachment and proliferation characteristics.

Fluorescence microscopy time-course analysis spanning 7 days (Days 1, 3, 5, and 7) demonstrated successful TIME-GFP cell attachment and progressive spatial colonization across the nanofiber architecture. Cells maintained characteristic endothelial morphology and robust GFP expression throughout the culture duration, indicating sustained cell viability and phenotypic stability on the framed substrates. The green fluorescent protein expression enabled precise visualization of viable endothelial cells and revealed dynamic network formation patterns across different experimental conditions.

Quantitative assessment revealed enhanced cellular density and improved spatial distribution with extended processing protocols, particularly evident in the 48 h treatment groups. Additionally, superior cell attachment characteristics were observed in the resin-side-up configuration, where the nanofiber surface was directly exposed to cellular contact, facilitating optimal cell–substrate interactions and subsequent proliferation dynamics.

### 3.5. Post-Processing Demonstration: Gold Coating Proof-of-Concept

**Figure 6:**
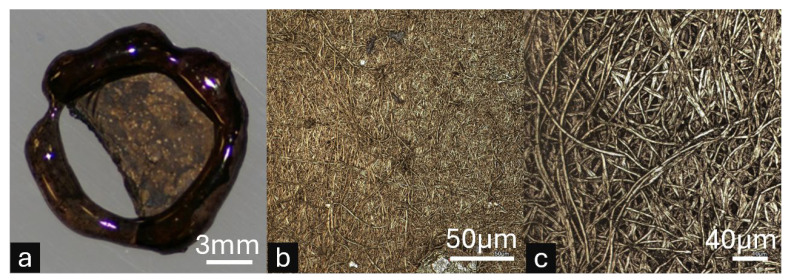
Proof-of-concept electroless gold plating on framed electrospun nanofiber membranes (ESNFMs) demonstrating post-processing capabilities. Note: This demonstration utilized preliminary JB Weld epoxy frames during method development, prior to Biotough D90 MF optimization. (**a**) Macroscopic view of gold-coated framed ESNFM showing successful metal deposition across the nanofiber surface, with localized membrane detachment from frame support illustrating mechanical stress limitations of the preliminary epoxy system. (**b**) High-magnification microscopic image revealing uniform gold coating distribution. (**c**) High-magnification microscopic image showing individual nanofibers, confirming successful metal nucleation and growth on the complex three-dimensional fibrous architecture. This proof-of-concept validates that properly supported ESNFMs can withstand aggressive multi-step chemical processing required for advanced functionalization.

To validate the functionality and post-processing capabilities of framed ESNFMs, electroless gold plating was performed as a representative complex surface modification procedure, with the results documented in Figure 6. This proof-of-concept investigation demonstrates the potential for advanced functionalization strategies that would be impractical or impossible with unsupported nanofiber membranes due to their inherent fragility during multi-step chemical processing.

The electroless gold coating protocol successfully achieved uniform metal deposition across the three-dimensional nanofiber architecture while maintaining overall membrane structural integrity (Figure 6a). Macroscopic examination revealed successful gold nucleation and coating distribution, though localized membrane detachment from the preliminary JB Weld epoxy frame support was observed, highlighting mechanical stress limitations of the initial framing system during electrochemical processing. This detachment provides valuable insight for optimizing frame–membrane adhesion in future iterations.

High-magnification microscopic analysis confirmed uniform gold coating distribution on individual nanofibers, demonstrating successful metal nucleation and growth across the complex fibrous surface topology (Figure 6b). The preservation of nanofiber morphology throughout the multi-step plating process validates that properly supported ESNFMs can withstand aggressive chemical treatments, opening pathways for advanced surface functionalization strategies, including conductive coatings, catalytic layers, and antimicrobial treatments that could significantly expand the functional capabilities of electrospun membrane systems.

## 4. Discussion

### 4.1. Processing Parameter Optimization and Biocompatibility Enhancement

The biocompatibility enhancement achieved through extended UV-curing protocols demonstrates substantial improvements in resin polymerization beyond conventional processing standards. The systematic improvement across multiple assessment methodologies suggests that standard 24 h protocols may result in incomplete crosslinking networks, with residual oligomers potentially contributing to reduced cellular viability rather than acute cytotoxicity. Extended curing protocols appear to enhance crosslink density while reducing extractable components, thereby optimizing surface chemistry for improved cellular compatibility. The quantified processing enhancement (meta-effect size = 1.08) provides a robust foundation for evidence-based manufacturing protocols.

The intermediate viscosity characteristics of Biotough D90 MF represent a critical design trade-off that directly impacts material compatibility. While this viscosity profile causes some resin spreading before UV curing (contributing to the observed dimensional variance), it enables excellent penetration through the fibrous matrix, ensuring complete mat embedding and uniform resin integration. Materials with poor wetting characteristics relative to the resin, such as highly hydrophobic polymers or chemically incompatible systems, may present integration challenges due to insufficient penetration of the fibrous matrix. Our preliminary investigations using conventional fused filament fabrication revealed that PEO-containing ESNFMs were incompatible with thermoplastic deposition methods, validating the necessity of our UV-DIW approach for this material system.

Notably, the 48 h extended processing protocol yielded a normalized viability of 116.5 ± 12.2%. While initially perplexing, this result is readily explainable by the mechanism of the Cell Counting Kit-8 (CCK-8) assay used in this study. The assay’s reagent, WST-8, is reduced by cellular dehydrogenases to produce a colored formazan product; the resulting absorbance is therefore a measure of total metabolic activity, not a direct count of viable cells. Because the assay measures metabolic rate, any substance in the test extract that stimulates this activity can lead to a result greater than 100%.

The most likely biological explanation for such a stimulatory effect is hormesis—a biphasic dose–response where low concentrations of a substance are stimulatory, while high concentrations are inhibitory [43]. In this context, the causative agents are trace chemical leachables that migrate from the resin. The extended 48 h post-processing protocol likely reduces these leachables from overtly toxic concentrations to the low, sub-toxic levels that trigger a mild, stimulatory hormetic response. Crucially, the magnitude of hormetic stimulation is typically modest, with a maximum of 30–60% above the control value [43]. The 16.5% increase observed here falls squarely within this established range, providing strong biological plausibility.

While this result does not reach the formal threshold for statistical significance when compared to a 100% baseline (one-sample *t*-test, *p* ≈ 0.073), the combination of a known assay mechanism susceptible to metabolic stimulation, a quantitatively consistent biological explanation in hormesis, and a suggestive statistical trend provides a robust interpretation. This is further supported by studies showing that leachables from polymers can significantly influence cytotoxicity outcomes [44]. Therefore, we conclude that the resin, when processed optimally, is not only non-cytotoxic but creates a favorable bioactive environment.

### 4.2. Direct Contact Performance and Material-Specific Cellular Responses

The differential biocompatibility observed between direct contact and extract testing methodologies reflects established patterns in cellular interactions with three-dimensional nanofibrous architectures. This phenomenon, documented across multiple studies, provides insights into the distinct cellular adaptation processes required for nanofiber substrates compared to conventional material surfaces. Recent investigations have further emphasized that “in vitro experimental conditions and tools can influence the safety and biocompatibility results of antimicrobial electrospun biomaterials” [45], highlighting the critical role of testing methodology in accurate biocompatibility assessment.

#### 4.2.1. Influence of Substrate Mechanics and Geometry on Cellular Adaptation

When cells encounter nanofiber environments, they undergo a characteristic adaptation phase involving cytoskeletal reorganization and focal adhesion maturation. This process includes “plasma membrane remodeling … along … fibers, by a physical mechanism”, termed one-dimensional membrane wetting [46], representing a fundamental difference from interactions with flat surfaces. The temporal dynamics of this adaptation are critical for interpreting biocompatibility results, with studies indicating that “at least 48 h is required for the cells to adhere to the surface” [47] of novel biomaterials. The observed pattern of initial morphological alterations followed by recovery to a “normal morphological state at later time points” suggests that reduced cell counts in short-term assays may reflect ongoing adaptation rather than cytotoxicity. This interpretation is supported by findings that “proliferation generally was inhibited on the nanofibrous scaffolds” [48] and that “low cell-counts on biomaterial samples is not necessarily an indication of resin cytotoxicity” [49].

These principles of cellular adaptation and mechanosensing directly explain the significant difference in cellular response observed between the disk and ring formats in this study (78.2% vs. 54.3% viability, respectively). The two formats present distinct mechanical environments. The solid disk format, with its continuous resin backing, presents the ESNFM as a textured surface on a rigid substrate. In this configuration, cells can establish robust focal adhesions and generate the cytoskeletal tension required for proliferation, similar to growing on standard tissue culture plastic. In contrast, the ring format creates a suspended, compliant scaffold. The inherent flexibility and low elastic modulus of the nanofiber mesh mean that cells encounter not only a complex 3D topography but also a lack of mechanical resistance. This high compliance can inhibit efficient cell spreading and the maturation of focal adhesions, a phenomenon known to slow the adaptive processes required for colonization and proliferation [50]. Therefore, the superior performance of the disk format is due to it providing a mechanically stable substrate that facilitates conventional cell adhesion, while the suspended ring format presents a more complex mechanobiological challenge.

#### 4.2.2. Material System Compatibility and Integration Mechanisms

The success of this framing approach depends critically on resin–substrate wetting and penetration dynamics. The chitosan–PEO system demonstrates favorable compatibility due to several factors: the hydrophilic nature of both polymers facilitates resin penetration, the optimized 94:6 ratio provides sufficient PEO content for electrospinnability while minimizing dimensional instability, and the intermediate viscosity of Biotough D90 MF enables thorough matrix infiltration without excessive spreading. Extension to alternative polymer systems, however, must be guided by the fundamental principle of matching the resin’s properties to the substrate. For materials with lower surface energy, such as PCL or PLGA, achieving optimal wetting may require the selection of resins specifically formulated to have a lower surface tension, for which commercial options and additives are available. Though sparse, the heterogeneous penetration patterns observed in SEM analysis (Figure 4f–h) underscore the complexity of the infiltration process, reflecting the intricate balance of factors including the material’s intrinsic surface energy, the presence of any surface impurities, and the physical architecture of the mat, such as local variations in porosity and tortuosity. This highlights that while the framing method is a powerful tool, its application to each new material system may require systematic optimization to ensure compatible resin–substrate interactions and achieve reliable integration.

### 4.3. Methodological Specifications and Design Capabilities

The experimental validation establishes key parameters for reproducible ESNFM framing across diverse applications. UV exposure duration emerged as a critical variable, with extended protocols exceeding 24 h demonstrating superior biocompatibility performance compared to standard curing approaches. The methodology accommodates various intensity parameters while maintaining material integrity, and the post-processing protocols, including washing and disinfection procedures, provide consistent biocompatibility outcomes.

The observed dimensional variance between target specifications (13 mm) and fabricated parts (15 mm) reflects the complex interplay between resin rheology and processing kinetics. This ~15% dimensional expansion results from resin spreading during the temporal gap between deposition and UV curing—a characteristic inherent to our sequential processing approach. For applications demanding precise dimensional tolerances, compensation strategies such as pre-scaling digital models by 0.87× can achieve target dimensions, though this approach may compromise feature resolution at smaller scales. The fundamental trade-off between dimensional accuracy and resin viscosity represents a critical design consideration: lower viscosity formulations that might reduce spreading would potentially compromise both the structural integrity of thin frame features and the essential penetration characteristics required for proper ESNFM integration.

The demonstrated fabrication capabilities through five-by-five frame arrays (25 samples in ~5 min) highlight the inherent scalability of this additive approach. The 3D printing-based methodology enables implementation of complex geometries—including variable frame widths, multi-aperture configurations, and anatomically relevant shapes—through straightforward digital model modifications. This design freedom positions the framing methodology as an adaptable platform technology, enabling rapid prototyping and customization for diverse tissue engineering applications beyond the circular frames validated in this study.

### 4.4. Practical Framework for ESNFM Handling and Testing Applications

This stabilization approach transforms electrospun nanofiber meshes from mechanically fragile materials into robust platforms suitable for standardized testing and characterization. The validated methodology addresses critical handling limitations that have historically complicated ESNFM implementation in research and development settings.

However, recognizing that many research laboratories lack access to integrated UV-curing print heads or specialized DIW systems, we developed two complementary approaches that leverage UV-curable resin benefits while accommodating equipment limitations. Our extrusion-based printing method employs layer-by-layer deposition with intermittent UV curing between layers, while our manual stamping technique provides a printer-free alternative. Both methods experience some degree of resin spreading due to the temporal gap between deposition and curing—a phenomenon reflected in our observed ~15% dimensional variance from target specifications. Despite this limitation, the approaches successfully transform delicate nanofiber mats into robust, easily handleable components through in situ frame fabrication that ensures strong adhesion and integration with the ESNFM.

#### 4.4.1. Mechanical Handling Improvements

The resin-stabilized ESNFMs demonstrate enhanced mechanical integrity that enables reproducible sample preparation across experimental replicates. The establishment of standardized geometries in both disk and ring formats provides consistent testing protocols while reducing variability in cellular assays previously attributed to material deformation. This mechanical stabilization also enables compatibility with automated handling systems, addressing a significant barrier to high-throughput applications.

#### 4.4.2. Testing Protocol Standardization

The biocompatibility validation across multiple configurations provides practical guidelines for ESNFM characterization. Direct contact assays revealed that disk formats demonstrated superior performance, likely due to optimized surface area presentation and reduced edge effects. Extract testing showed that all formats exceeded Grade 0 classification standards, validating the fundamental biocompatibility of the stabilized materials. These format-dependent variations align with recent findings demonstrating that experimental conditions and methodological choices can significantly influence biocompatibility outcomes for electrospun materials [45]. These findings emphasize the importance of considering temporal factors, with extended assessment timeframes recommended to account for the cellular adaptation phases characteristic of three-dimensional nanofibrous substrates.

#### 4.4.3. Integration with Existing Workflows

This stabilization technology enables the integration of ESNFMs into established biofabrication workflows where material stability is critical. The proof-of-concept electroless gold coating demonstrates this enhanced durability. This aggressive, multi-step chemical process, which an unsupported ESNFM cannot survive, was made possible by the mechanical support of the frame. It is important to note that this specific demonstration was a preliminary experiment conducted with a non-optimized epoxy resin to validate the framing concept itself. The performance of the final, biocompatible Biotough D90 MF resin was not evaluated in this specific chemical process. Nonetheless, the experiment successfully shows that the framing approach transforms fragile membranes into robust components capable of withstanding complex post-processing. This allows for the potential development of advanced functionalized platforms, such as conductive scaffolds for biosensing, and enables researchers to focus on optimizing biological functionality rather than managing material handling limitations.

### 4.5. Limitations and Future Directions

While this study demonstrates a successful method for ESNFM stabilization, several limitations merit consideration and guide future work. First, the observed ±15% dimensional variance may require refinement for applications demanding higher geometric precision. This could be addressed through software-based compensation algorithms or, more fundamentally, through the rheological optimization of resin formulations to balance dimensional accuracy with mechanical properties. Second, this study did not include quantitative mechanical testing to assess how properties like tensile strength or modulus are affected by different UV curing durations. While no functional differences in durability were observed during routine handling, formal mechanical characterization would provide valuable data for optimizing the frames for more mechanically demanding applications. Finally, we demonstrated this framing technique with only one membrane material. Chitosan has a medium, though variable [51], total surface energy of 30–40 mJ/m^2^, which promoted infiltration of the resin through its fibrous matrix. Infiltration of the resin through other materials with lower surface energies may be limited, reducing the effectiveness of the technique. Notably, the surface energy of a material can change considerably due to the electrospinning method, despite identical polymer working solutions [52].

Future investigations should prioritize the following: (1) validating this framing technique across a broader range of electrospun polymers, especially low surface energy materials to establish its range of applicability; (2) expansion to multi-layer membrane architectures for more complex tissue models; and (3) integration of these stabilized membranes into dynamic culture systems, such as microfluidic organ-on-chip platforms.

## 5. Conclusions

We have successfully developed and validated a novel framing technique that transforms fragile electrospun nanofiber membranes into robust, biocompatible platforms suitable for advanced tissue engineering applications. Both 3D printing and manual stamping methods effectively create integrated support frames on chitosan–PEO ESNFMs using UV-curable Biotough D90 MF resin. Extract testing demonstrates exceptional biocompatibility (116.5% normalized response with optimized processing), while direct contact assessment achieves acceptable performance (78.2% optimal configuration). Extended UV-curing protocols (48 h vs. 24 h) systematically enhance biocompatibility across all assessment methodologies, and the framed ESNFMs withstand complex post-processing procedures while maintaining structural integrity.

This enabling technology addresses critical handling limitations that have constrained widespread adoption of ESNFMs in advanced biofabrication applications, facilitating their integration into standardized research protocols and clinical translation pathways. The quantitative biocompatibility validation and processing optimization provide the foundation for scalable manufacturing of ESNFM-based tissue engineering platforms. The immediate next steps are as follows: (1) resin formulation optimization targeting 95% dimensional accuracy while maintaining biocompatibility, (2) validation across expanded polymer systems to demonstrate universal applicability, (3) development of automated fabrication protocols for GMP-compliant production, and (4) integration demonstrations in microfluidic organ-on-chip platforms. These advances position framed ESNFMs as standardized components for next-generation tissue engineering applications.

## 6. Patents

Parts of this work may be covered by pending patent applications.

## Figures and Tables

**Table 1:** Three-dimensional printing parameters for resin UV-curing process.

Parameter	Value	Description/Notes
Layer Height	0.1 mm	Resin layers alternated with 0.1 mm UV-curing layers
Extrusion Multiplier	2	Compensation for non-material-depositing UV layers
Effective Resin Layer Thickness	0.2 mm	Resulting from alternating deposition/curing sequence
Total Layer Count	4 layers	Achieved 0.8 mm total specimen height
UV Pen Power	20%	Optimized for adequate curing without LED overheating
Slicer Configuration	Alternating mode	Synchronized syringe extruder (resin) and UV pen (curing) operation

Note: The printing process employed an alternating extruder sequence where each resin deposition layer was immediately followed by UV curing before proceeding to the next layer.

**Table 2:** Summary of biocompatibility assessment results.

Assessment Type	Processing Protocol	Configuration	Normalized Response (%)	Biocompatibility Grade *	Statistical Significance
**Extract Testing**	24 h Standard	---	90.3 ± 3.5	Grade 0 (Excellent)	*p* < 0.001 vs. 70% threshold
	48 h Extended	---	116.5 ± 12.2	Grade 0 (Excellent)	*p* < 0.001 vs. 70% threshold
**Direct Contact**	24 h Standard	Overall	49.8 ± 15.5	Grade 3 (Poor)	*p* = 0.062 vs. 70% threshold
	48 h Extended	Overall	67.9 ± 28.1	Grade 2 (Marginal)	*p* = 0.421 vs. 70% threshold
	48 h Extended	Disk Format	78.2 ± 32.4	Grade 1 (Acceptable)	*p* = 0.267 vs. 70% threshold
	48 h Extended	Ring Format	54.3 ± 13.8	Grade 3 (Poor)	*p* = 0.108 vs. 70% threshold

* Biocompatibility grades based on ISO 10993-5 standards: Grade 0 (>90%), Grade 1 (70–90%), Grade 2 (50–70%), Grade 3 (<50%). Data presented as mean ± standard deviation.

## Data Availability

The original contributions presented in this study are included in the article/Supplementary Materials. Further inquiries can be directed to the corresponding author.

## Supplementary Materials

The following supporting information can be downloaded at https://www.mdpi.com/article/10.3390/mi16080887/s1, List S1: Prusa “filament” custom gcode; File S1: 13mmx1.6Wx1H_circle.3mf; File S2: Frame stamp final.stl; File S3: Revised SIMPoly code fitting a lognormal distribution.

## Abbreviations

The following abbreviations are used in this manuscript:

3D	Three-Dimensional
ANOVA	Analysis of Variance
BBB	Blood–Brain Barrier
CCK-8	Cell Counting Kit-8
CI	Confidence Interval
COC	Cyclic Olefin Copolymer
CS	Chitosan
CV	Coefficient of Variation
ECM	Extracellular Matrix
ESNFM	Electrospun Nanofiber Membrane
FDA	Food and Drug Administration
FFF	Fused Filament Fabrication
GFP	Green Fluorescent Protein
HSD	Honestly Significant Difference
ID	Inner Diameter
IPA	Isopropyl Alcohol
ISO	International Organization for Standardization
OD	Outer Diameter
OOC	Organ-on-Chip
PBS	Phosphate-Buffered Saline
PDA	Polydopamine
PEO	Polyethylene Oxide
PLA	Polylactic Acid
PVC	Polyvinyl Chloride
SEM	Scanning Electron Microscopy
STL	Stereolithography
TIME-GFP	Telomerase-Immortalized Human Microvascular Endothelial Cells Expressing Green Fluorescent Protein
UV	Ultraviolet
VBA	Visual Basic for Applications
