# Supplementary material for: 3D-Printed Biocompatible Frames for Electrospun Nanofiber Membranes: An Enabling Biofabrication Technology for Three-Dimensional Tissue Models and Engineered Cell Culture Platforms
Source: Micromachines (Basel). 2025 Jul 30;16(8):887. doi: 10.3390/mi16080887 (PMC12388470; doi:10.3390/mi16080887)
Supplement: Supplementary file 1 [file micromachines-16-00887-s001.zip › List S1.docx]

Supplementary material.

Prusa “filament” custom gcode

- SDS5 Syringe Extruder
  - Start G-Code

; Filament gcode

M229 E01 D0; turns on E-values (shoudl disable hyrel hardware flow rate)

M721 S10000 E300 P80 T12 - ;sets the hardware UNPRIME values, in rate (S), min dwell time (E), and pulses (P) for slot 2 (T)

M722 S10000 E300 P105 T12 - ;sets the PRIME values as above;

M221 S1.0 T12 P85 W1.6 Z0.1 ;sets hyrel hardwarea flow rate

M722 S1000 E1000 P2000 T1 I1 ; prime sds

- - End G-Code

;Filament-specific end gcode

M229 E1 D1; turns off evalues

M721 S1000 E100 P-2000 T1 I1; retract sds

- UV Pen
  - Start G-Code

; Filament gcode

;http://hyrel3d.com/wiki/index.php/UV_and_Clench#UV_Pen

M229 E0 D0

M703 T14 S11

M620 T14 E1

M621 T14 P20

- - End G-Code

; Filament-specific end gcode

M620 T14 E0

M229 E1 D1 S0.02;
